# Lidocaine transdermal patches reduced pain intensity in neuropathic cancer patients already receiving opioid treatment

**DOI:** 10.1186/s12904-023-01126-3

**Published:** 2023-01-07

**Authors:** Jui-Hung Tsai, I-Ting Liu, Pei-Fang Su, Ying-Tzu Huang, Ge-Lin Chiu, Yu-Yeh Chen, Wei-Shu Lai, Peng-Chan Lin

**Affiliations:** 1 Department of Oncology, National Cheng Kung University Hospital, College of Medicine, National Cheng Kung University, No. 138, Sheng-Li Road, Tainan, Taiwan; 2 Department of Internal Medicine, National Cheng Kung University Hospital, College of Medicine, National Cheng Kung University, Tainan, Taiwan; 3 Center for Hospice Palliative Shared Care, National Cheng Kung University Hospital, College of Medicine, National Cheng Kung University, Tainan, Taiwan; 4 Department of Statistics, National Cheng Kung University, Tainan, Taiwan; 5 Department of Nursing, National Cheng Kung University Hospital, College of Medicine, National Cheng Kung University in Tainan, Tainan, Taiwan; 6 Department of Nursing, College of Medicine, National Cheng Kung University, Tainan, Taiwan

**Keywords:** Neuropathic pain, Transdermal patch, Cancer pain, Numerical rating scale, Analgesic

## Abstract

**Background:**

Limited efficacy has been observed when using opioids to treat neuropathic pain. Lidocaine patches reduce neuropathic pain in postherpetic neuralgia, but their benefits for cancer-related neuropathic pain remain unclear. This study aimed to investigate a treatment for cancer-related neuropathic pain.

**Methods:**

We conducted a prospective, open-label, single-arm study to assess the efficacy and safety of lidocaine transdermal patches in patients experiencing localized, superficial, neuropathic cancer pain. Terminal cancer patients already receiving opioid treatment participated in the 3-day study. The primary endpoint was pain intensity evaluated by the numerical rating scale (NRS). The secondary endpoints were the pain relief score and the quality of analgesic treatment.

**Results:**

The results showed a significant difference in the median NRS over 3 days (Kruskal–Wallis test, *p* < 0.0001). The median NRS pain intensity from Day 1 to Day 3 was 4.0 with 95% C.I. (3.3, 5.0), 3.0 (2.5, 3.5), and 2.6 (2.0, 3.0), respectively. The difference between the median NRS pain intensities of any 2 days was significant (Wilcoxon signed-rank test, *p* < 0.0001). The generalized estimating equation (GEE) estimation model showed significant differences between the NRS pain intensities on any 2 days. There was no significant difference in the pain relief score or the quality of analgesic treatment.

**Conclusions:**

In this study, the 5% lidocaine transdermal patch reduced the NRS pain intensity in neuropathic cancer patients already receiving opioid treatment. Treatment of localized and superficial neuropathic pain caused by cancer was well tolerated and effective.

**Supplementary Information:**

The online version contains supplementary material available at 10.1186/s12904-023-01126-3.

## Background

Although opioids are effective and safe in chronic cancer pain treatment, neuropathic pain (NP) is a common problem encountered in 40% of cancer patients [1–3]. A cancer patient with NP has abnormal sensitivity to either noxious (hyperalgesia) or innocuous (allodynia) stimuli at the site of pain resulting from cancer insults to the peripheral or central nervous systems [4, 5]. Light clothing, flowing water, or even cold air may cause discomfort to these patients. Analgesics such as opioids provide some pain relief but are less effective for NP than for nociceptive pain. Nonsteroidal anti-inflammatory drugs and other adjuvants are also ineffective in treating neuropathic pain. It is usually described as “intractable” and “opioid-resistant” [6].

There are several classes of pain medications that provide moderate relief, but they are unlikely to provide complete or near-complete relief. While antidepressants and anticonvulsants effectively treat neuropathic pain, adverse effects may prevent adequate analgesia. As an aminoethylamide local anesthetic, lidocaine inhibits voltage-gated sodium channels on excitable membranes, preventing nerve impulses from being generated and conduction [7, 8]. A topical lidocaine patch can relieve various neuropathic pain conditions after surgery [1, 9]. Several neuropathic symptoms, such as numbness, tingling, and pain, can be reduced with a lidocaine patch [10]. Lidocaine patch application duration and the surface area covered determine how much is absorbed. There is minimal systemic absorption of lidocaine from the patch. Lidocaine applied to intact painful skin produces significant pain relief without clinically significant lidocaine serum levels [7, 8]. Patch application relieves pain caused by damaged nerves and is well tolerated.

In March 1999, the Food and Drug Administration (FDA) approved a 5% lidocaine patch to relieve postherpetic neuralgia. However, it is unknown whether a lidocaine patch effectively treats NP cancer patients who are already receiving opioid treatment. The present study aimed to investigate the analgesic effect of a 5% lidocaine patch in patients with neuropathic cancer pain in hospice care. NP was assessed by pain intensity via the numerical rating scale, pain relief score, and analgesic treatment quality [11–13]. The safety of lidocaine patches in NP cancer patients was also evaluated.

## Methods

### Demographic characteristics

We conducted a prospective, open-label, single-arm study for pain control. A total of 96 terminal cancer patients with stage IV (advanced) malignant disease who were admitted to the hospice ward at National Cheng Kung University Hospital (NCKUH), were enrolled. The eligibility criteria were 18 years of age and above, male or female, who were treated with opioids for cancer-related pain and still experiencing neuropathic cancer pain evaluated by the LANSS Pain Scale [14]. In addition to neuropathic pain, the pain had to be well localized, superficial, and involve positive symptoms such as allodynia, raised pin-prick threshold, and hyperalgesia. Regardless of the intensity, all patients with such neuropathic pain were included. There was no contraindication to topical anesthetic application. The study period was 3 days, and all participants signed informed consent forms. Patient symptoms were evaluated by questionnaires, including an 11-point pain intensity scale, a 5-item pain relief score, and 5-item analgesic treatment quality. The exclusion criteria included the following: (1) skin lesions with bacterial infection and allergies to para-aminobenzoic acid derivatives (procaine, tetracaine, benzocaine, etc.); (2) a significant concomitant illness that the investigator believed would interfere with the evaluation of the study medications, including lidocaine, tocainide, mexiletine, and phenytoin; and (3) the patient received treatment with topically applied medication (e.g., lidocaine/prilocaine cream, capsaicin cream, and doxepin cream) 72 hours before the study. The National Cheng Kung University Hospital institutional review board approved this study (BR-100-005-C).

### Treatment schedule

**Fig. 1:**
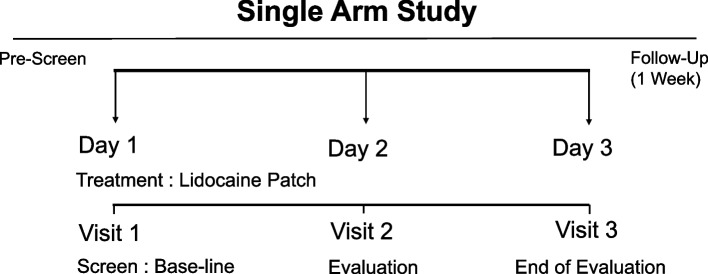
Treatment schedule. The first visit included screening and enrollment; eligible subjects received the study patch for 3 days. Subsequently, the second visit was scheduled on the second and third days of the study, and the third visit was the end of the evaluation

In this study, the first visit included screening and enrollment, and eligible subjects (with neuropathic cancer pain) received the study patch for 3 days (Fig. 1). Subsequently, the second/third visit was scheduled on the second or third day of the study for endpoint evaluation. All subjects were assigned to the lidocaine group for 3 days. Pain intensity, including the numerical rating scale (NRS)-resting score, was also assessed as baseline data before patch application. The enrolled subjects applied the patch to well-defined, intact skin to cover the most painful area. Subjects were allowed to use up to three patches simultaneously, each for 12–24 hours, once to twice daily. Any observed and spontaneously reported adverse events (AEs) were recorded. In addition to collecting information on concurrent diseases and medications, the investigator evaluated clinical efficacy and safety at each visit. To assess the outcome of ongoing adverse events, the subject was contacted within 1 week after the trial ended for a follow-up. We also reported any new adverse events that occurred during this period.

### Assessments of clinical efficacy

The primary endpoint of the analgesic effect of the experimental patch was evaluated by the 11-point NRS pain intensity on Day 2 and Day 3. The secondary endpoints of efficacy were evaluated by the five-item pain relief score and analgesic treatment quality on Day 2 and Day 3. Pain intensity was evaluated using a horizontal line, with a total of 11 points, ranging from 0 to 10, on a numerical rating scale (NRS) [11, 12], which interprets the severity of pain on a scale ranging from 0 = “no pain” to 10 = “the worst pain imaginable.” The subject was questioned about the NRS-resting score on Day 1. Furthermore, the NRS score for neuropathic pain was recorded in parallel. The pain intensity was assessed from before the first patch was applied as the baseline (on Day 1) to after the last patch was applied (on Day 2 and Day 3). The 5-item pain relief score was determined as follows. Pain relief was assessed using a category scale consisting of the following 5 scores: 0 = “no” pain relief; one = “slight” pain relief; two = “moderate” pain relief; three = “much” pain relief; and four = “complete” pain relief [1]. A modified version of the Brief Pain Inventory (BPI) [15] was used as an assessment tool for analgesic treatment quality. The subject rated the 5-item quality of the analgesic treatment using the following five-item scale: 1 = excellent, complete pain relief, performance status reaching 100%, no compromise of sleep and appetite; 2 = good, tolerable pain not longer than half an hour or disappearance of pain but with compromised sleep, appetite, or performance status; 3 = satisfactory, tolerable mild degree of pain, no further medication requirement; 4 = insufficient, feeling better but with pain control that was not adequate, apparent pain sensation; and 5 = poor, no improvement at all, even worse pain.

### Assessments of safety

We evaluated the safety of the lidocaine patches using vital signs, skin inspections, and skin examinations. The application area affected by the lidocaine patch was recorded. Under the same conditions, systolic blood pressure (SBP), diastolic blood pressure (DBP) and heart rate (HR) were measured. A brief examination of the skin under the patches was carried out at each visit to document skin redness, blanching, or irritation. Detailed adverse event data were recorded by the patient and summarized.

### Statistical analysis

The demographic data and the distribution of each variable were described using frequency distributions. Data analyses included descriptive and inferential statistics. A descriptive analysis was conducted, including the estimated mean and standard deviation for continuous variables and the percentages and frequencies for categorical variables. Because of violations of the normality assumption, the Wilcoxon signed-rank test and the Kruskal–Wallis test were used to compare whether the median values of the two/three groups were equal. To account for the correlation among repeated measures from the same cancer patient, a generalized estimating equation (GEE) was used to estimate the effect of covariates on the mean of the response variables. All statistical tests were 2-sided, with a *p value* less than 0.05 considered to indicate statistical significance. Safety parameters such as vital signs and skin inspection were also summarized and displayed with descriptive summary statistics. The analyses were performed using the R 4.0.2 version software package for Windows.

## Results

### Patient characteristics

A total of 96 terminal cancer patients in hospice wards were referred to our studies. The median age was 58 years old, and 50/46 patients were male/female. The most common types of cancer were as follows: head and neck cancer (HNSCC), 15 (15.6%); upper gastrointestinal (UGI) cancer, 14 (14.6%); colorectal cancer (CRC), 12 (12.5%); genitourinary (GU) cancer, 10 (10.4%); lung cancer, 10 (10.4%); breast cancer, 10 (10.4%); and hepatocellular carcinoma (HCC), 9 (9.4%). The cancer subtypes and patient characteristics are listed in Table 1 and Supplementary Table 1. In addition to opioid therapy, some patients received nonsteroidal anti-inflammatory drugs (NSAIDs), acetaminophen, corticosteroids, and antidepressants as analgesics. Lidocaine patches were applied on the torso (54.2%), legs (20.8%), head and neck (18.8%), arms (5.2%), and hands (1.0%). Among the sensory dysfunctions associated with neuropathic pain were allodynia (54.2%), an elevated pin-prick threshold (46.7%), hyperalgesia (32.3%), tingling (35.4%), pin-pricks (31.3%), stabbing (29.2%), numbness (27.1%), tightness/stretching (34.3%), burning (20.8%), and cutting/laceration (29.2%). The percentages for the baseline NRS pain intensity at rest were 39 (40.6%) patients with 0–3 points, 50 (52.1%) patients with 4–7 points, and 7 (7.3%) patients with 8–10 points. The percentages for the baseline NRS pain intensity on movement were 8 (8.3%) patients with 0–3 points, 53 (55.2%) patients with 4–7 points, and 35 (36.5%) patients with 8–10 points.

**Table 1:** Characteristics of 96 cancer patients with neuropathic pain

Characteristics	Number (%)
Patient No.	96 (100)
Age (years)	
Median age, yr (Range)	58 (20-96)
Performance Status	
1-2	20 (20.8)
3-4	76 (75.2)
Sex	
Male/Female	50/46 (58.3/41.7)
Diagnosis	
HNSCC	15 (15.6)
UGI Cancer	14 (14.6)
Colorectal Cancer	12 (12.5)
GU Cancer	10 (10.4)
Lung Cancer	10 (10.4)
Breast Cancer	9 (9.4)
HCC	9 (9.4)
GYN Cancer	6 (6.3)
Thyroid Cancer	6 (6.3)
Sarcoma	3 (4.1)
Lymphoma	1 (1.0)
MUO	1 (1.0)
Concurrent analgesic regimen	
Opioids	96 (100)
NSAID	15 (15.6)
Acetaminophen	15 (15.6)
Corticosteroid	14 (14.6)
Antidepressants	7 (7.3)
Pain score at rest (NRS)	
0-3	39 (40.6)
4-7	50 (52.1)
8-10	7 (7.3)
Pain score on movement (NRS)	
0-3	8 (8.3)
4-7	53 (55.2)
8-10	35 (36.5)
Applied Sites	
Torso	52 (54.2)
Legs	20 (20.8)
Head and Neck	18 (18.8)
Arms	5 (5.2)
Hands	1 (1.0)
Sensory Dysfunction	
Allodynia	52 (54.2)
Raised Pin-Prick Threshold	45 (46.9)
Hyperalgesia	31 (32.3)
Tingling	34 (35.4)
Pinprick	30 (31.3)
Stabbing	28 (29.2)
Numbness	26 (27.1)
Tight/stretched	33 (34.3)
Burning	20 (20.8)
Cutting/Laceration	28 (29.2)

*Abbreviations*: *HNSCC* Head and neck squamous cell carcinoma, *UGI* upper gastrointestinal, *GU* genitourinary, *HCC* hepatocellular carcinoma, *GYN* gynecological, *MUO* malignancy of unknown origin, *NRS* numerical rating scale

### Clinical efficacy

#### Pain intensity over three days

**Fig. 2:**
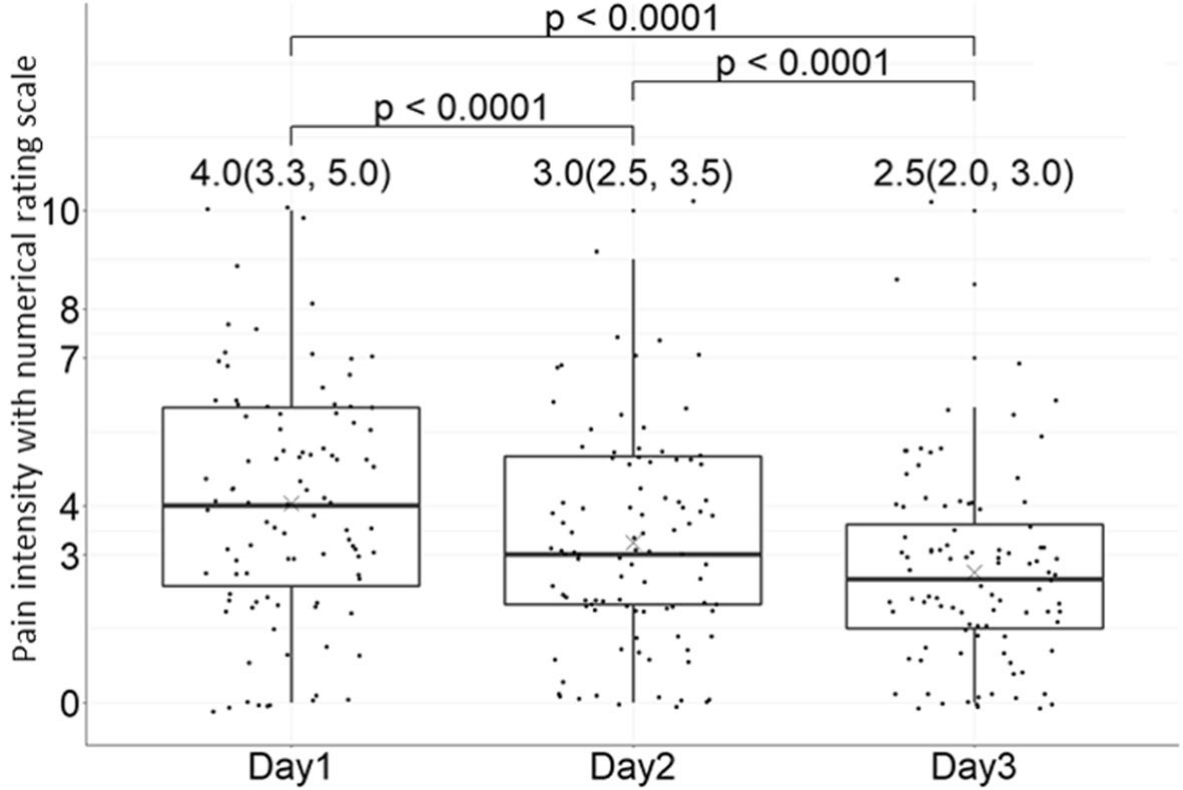
Effect on pain intensity assessed by the numerical rating scale. Pain intensity was assessed using a numerical rating scale (NRS), which interprets the severity of pain on a scale ranging from 0 (no pain) to 10 (worst pain imaginable). The pain intensity was assessed before the first patch was applied as the baseline (on Day 1) and after the last patch was used (on Day 3). The results showed a significant difference in the median NRS pain intensity over 3 days (*p value* < 0.0001)

We analyzed pain intensity on a 11-point scale using the Kruskal–Wallis test and Wilcoxon signed-rank test. The Kruskal–Wallis test showed significant differences in the median pain scores over 3 days (Supplementary Fig. 1, Kruskal–Wallis test, *p value* < 0.0001). The average and median pain scores demonstrated a decreasing trend with increasing use time. The median NRS pain intensity from Day 1 to Day 3 was 4.0 with 95% CI (3.3, 5.0), 3.0 (2.5, 3.5), and 2.6 (2.0, 3.0), respectively. The median NRS pain intensity of any 2 days was significant (Fig. 2. Wilcoxon signed-rank test, *p value* < 0.0001). We reported changes in the number of patients with different NRS pain intensities on any 2 days (Table 2 and Supplementary Table 2). On Day 1 and Day 2, 51 patients had decreased NRS pain intensity. A total of 8.3 and 44.8% of patients had a reduction of more than 4 points and 1–3 points, respectively. Sixteen patients had increased NRS pain intensity. A total of 14.6 and 2.1% of patients reported that their pain intensity increased by 1–4 points and 5 points, respectively. There were similar results for any 2 days. The data are shown in Table 2.

**Table 2:** Comparisons of posttreatment change scales for pain intensity

Pain intensity with numerical rating scale						
Decrease^a^				No Change^b^	Increase^a^	
	≥7	4-6	1-3	0	1-4	≥5
	N (%)	N (%)	N (%)	N (%)	N (%)	N (%)
Day 1-2	0 (0%)	8 (8.3%)	43 (44.8%)	29 (30.2%)	14 (14.6%)	2 (2.1%)
Day 1-3	2 (2.1%)	13 (13.5%)	43 (44.8%)	24 (25.0%)	13 (13.5%)	1 (1.0%)
Day 2-3	0 (0%)	6 (6.3%)	39 (40.6%)	40 (41.7%)	10 (10.4%)	1 (1.0%)

^a^Decrease (increase) was defined as a change in numerical rating scale (NRS) pain intensity of more than 1

^b^No change was defined as a change in numerical rating scale (NRS) pain intensity of less than 1

We also used the generalized estimating equation (GEE) model for the same patient with a three-day series assessed by NRS pain intensity (Table 3). The GEE estimation model shows that the NRS pain intensity on the second day decreased by approximately 0.8 points compared with the first day. On the third day, the NRS pain intensity decreased by approximately 1.4 points compared with the first day. The test results showed significant differences between the NRS pain intensities on any 2 days (Table 3). Based on the above results, the patch had a significant effect on the NRS pain intensity score.

**Table 3:** GEE model evaluation of pain intensity

	Estimate	Std	Wald	*p value*
Intercept	4.04	0.24	274.80	<0.0001
Day 2	-0.80	0.19	17.77	<0.0001
Day 3	-1.40	0.23	37.53	<0.0001

*Abbreviations*: *GEE* generalized estimating equation, *Std* standard deviation

#### Pain relief score

We evaluated the clinical impact of lidocaine analgesics over 3 days using a five-item pain relief score. The results showed no significant difference in the median of the three-day pain relief score. Supplementary Fig. 2 shows that the average and median pain relief scores did not change significantly (Supplementary Fig. 2A; Kruskal–Wallis test, *p value* = 0.79). According to the Wilcoxon signed-rank test (*p value* = 0.24, 0.36, 0.9), there were no significant differences between the median pain relief scores for any 2 days. Based on the GEE model, the patch had no significant impact on the pain relief score.

#### The quality of analgesic treatment

No significant difference was found in the quality of analgesic treatment. Supplementary Fig. 2 shows that the average and median analgesic treatment quality did not change significantly (Supplementary Fig. 2B; Kruskal–Wallis test, *p value* = 0.77). There were no significant differences in the median analgesic treatment quality for any 2 days as assessed by the Wilcoxon signed-rank test (*p value* = 0.21, 0.7, 0.029). Based on the GEE model, the patch had no significant impact on the quality of analgesic treatment score.

### Clinical adverse events

The side effects were observed in the 96 terminal cancer patients. Minimal adverse events were noted, including cold sensation (6.3%), irritation (3.1%), itching (3.1%), rash (3.1%), and redness (1.0%), as shown in Table 4.

**Table 4:** Adverse events in 96 cancer patients after the lidocaine patch

Characteristics	Number (%)
Patient No.	96 (100)
Adverse event	
Cold Sensation	6 (6.3)
Irritation	3 (3.1)
Itching	3 (3.1)
Rash	3 (3.1)
Redness	1 (1.0)

## Discussion

To treat neuropathic pain, we must select the appropriate approach, including the dosage and route of administration for hospice care. Most medications given to patients near the end of their lives are topical or subcutaneous [16]. In this open-label study, lidocaine topical medications were administered to hospice and cancer patients suffering from neuropathic pain. Based on the results, there are three main conclusions. (i) Topical lidocaine patches reduced pain intensity in terminal cancer patients with localized and superficial NP. (ii) Hospice cancer patients generally tolerated topical lidocaine well. (iii) Lidocaine patches had no clinically significant effect on pain relief scores or the quality of analgesic treatment. In this study, efficacy and safety were demonstrated in patients with neuropathic cancer who were already receiving opioid treatments, especially in hospice care.

Hans G et al. [17] reported a prospective study including 40 patients with severe neuropathic pain due to surgical or nonsurgical trauma. Approximately 52.5% of the patients benefited from treatment with lidocaine, with the severity of their pain on an NRS diminishing at the end of the study. Our study revealed that 51.1% (51/96) of patients had decreased pain intensity from Day 1 to Day 2, similar to a previous superficial neuropathic pain study. In a placebo-controlled crossover study, a 5% lidocaine medicated plaster effectively relieved ongoing pain and allodynia within the first 8 h, with effects lasting up to 1 week [18]. As in our results, a single arm and open-label study, the severity of the pain was analyzed using an NRS, thus measuring Day 1 to Day 3. Compared with double-blind trials, open-label trials may overestimate lidocaine patch effectiveness. In a previous study [18], the lidocaine patch significantly reduced pain on Day 3 of the 7-day treatment period. Our results showed that statistical significance was reached on Day 2 and Day 3 after the first patch application. We demonstrated that adding 5% lidocaine patches could be helpful in the short term for treating neuropathic cancer pain by decreasing the NRS pain intensity.

This study’s primary efficacy variable was the resting-pain intensity measured with a numerical rating scale (NRS). We planned to compare NRSs from any two groups using the Wilcoxon signed-rank test. To check whether the two medians were similar, if the true median difference between the two populations was set to 1 and the common standard deviation of the two populations was set to 1.5, the required minimum sample size was 67. In this setting, the type I error probability associated with this test of this null hypothesis was 0.05, and the study power was 0.95. As calculated by power calculations, we enrolled 96 patients with terminal cancer, which was greater than the 67 minimally required samples.

The Kruskal–Wallis test considers the data collected over 3 days as coming from independent and different individuals. A generalized estimating model is used to address the issue of other correlated values of the same patient at different time points since the same patient completed the pain measurement form for 3 days, which is thus repeated measurement data. The GEE estimation demonstrated statistical significance for the pain reduction effect as measured by the NRS pain intensity in the time series and the Kruskal–Wallis and Wilcoxon signed-rank tests, which showed statistical significance in NRS pain intensity for any 2 days.

Topical and local anesthetic patches are highly appealing therapies for allodynia due to their simplicity and safety, especially in hospice care. This appeal is based on the benefits of the patch formulation as a barrier to external stimuli and its lack of clinically significant systemic absorption, drug interactions, and severe side effects [7, 8]. This study showed that the investigated treatment is effective and carries no risk in different terminal cancer patients.

A significant difference in pain relief or analgesic treatment quality was not found in the GEE estimates. Additionally, Derry et al. reviewed topical lidocaine for neuropathic pain in adults [19]. There is no evidence to support the use of topical lidocaine for treating neuropathic pain from high-quality randomized controlled trials. The emotional dimension and quality of life were not assessed in our study. The present study failed to demonstrate pain relief and quality improvements with the lidocaine patch. Compared to an 11-point NRS pain intensity score, the patch did not significantly support a five-item pain relief or quality of analgesic treatment score.

There are certain limitations to extrapolating our results. This was an uncontrolled, open-label, single-arm study and is subject to a significant risk of the placebo effect and recall bias. A short study period and lack of long-term follow-up data also limit the study. The lack of a neuropathic pain-specific tool for the primary outcome might also be a limitation. We did not set a standard dose of lidocaine patches in this study. The opioid doses for participating patients were not well documented in our study. A lidocaine patch would unlikely penetrate deep enough to have a pharmacological effect unless the patient’s neuropathic pain was cutaneous or superficial.

## Conclusions

In this study, a 5% lidocaine skin patch reduced NRS pain intensity in patients with neuropathic cancer pain. It was well tolerated and effective for treating localized and superficial neuropathic cancer pain.

## Supplementary Information

**Additional file 1: Supplementary Table 1.** 96 Patients’ Characteristics. **Supplementary Table 2.** 96 patients with Pain score (Day 1–3: Pain intensity with numerical rating scale, Pain relief score, Analgesic treatment quality).

**Additional file 2: Supplementary Fig. 1.** The Kruskal–Wallis test showed significant differences in the median and mean pain scores over three days (*p* value < 0.0001).

**Additional file 3: Supplementary Fig. 2.** Effect on pain relief score and quality of analgesic treatment. A. a five-item pain relief score was assessed on Day 1, Day 2 and Day 3. The results showed no significant difference in the median of the three-day pain relief score (*p* value = 0.79). B. a five-item quality of analgesic treatment was assessed on Day 1, Day 2 and Day 3. The results showed no significant difference in the median of the three-day (p value = 0.77).

## Data Availability

The datasets used and analyzed during the current study are available from the supplementary information files of this manuscript.

## Abbreviations

AEs: Adverse events
CRC: Colorectal cancer
DBP: Diastolic blood pressure
FDA: Food and Drug Administration
GEE: Generalized estimating eq.
GU: Genitourinary
HCC: Hepatocellular carcinoma
HNSCC: Head and neck cancer
HR: Heart rate
NCKUH: National Cheng-Kung University Hospital
NP: Neuropathic pain
NRS: Numerical rating scale
NSAIDs: Nonsteroidal anti-inflammatory drugs
SBP: Systolic blood pressure
UGI: Upper gastrointestinal
WHO: World Health Organization
