# Supplementary material for: Lidocaine transdermal patches reduced pain intensity in neuropathic cancer patients already receiving opioid treatment
Source: BMC Palliat Care. 2023 Jan 7;22:4. doi: 10.1186/s12904-023-01126-3 (PMC9824981; doi:10.1186/s12904-023-01126-3)
Supplement: Supplementary file 2 — Additional file 2: Supplementary Fig. 1. The Kruskal–Wallis test showed significant differences in the median and mean pain scores over three days (p value < 0.0001). [file 12904_2023_1126_MOESM2_ESM.pptx]

## Slide 1
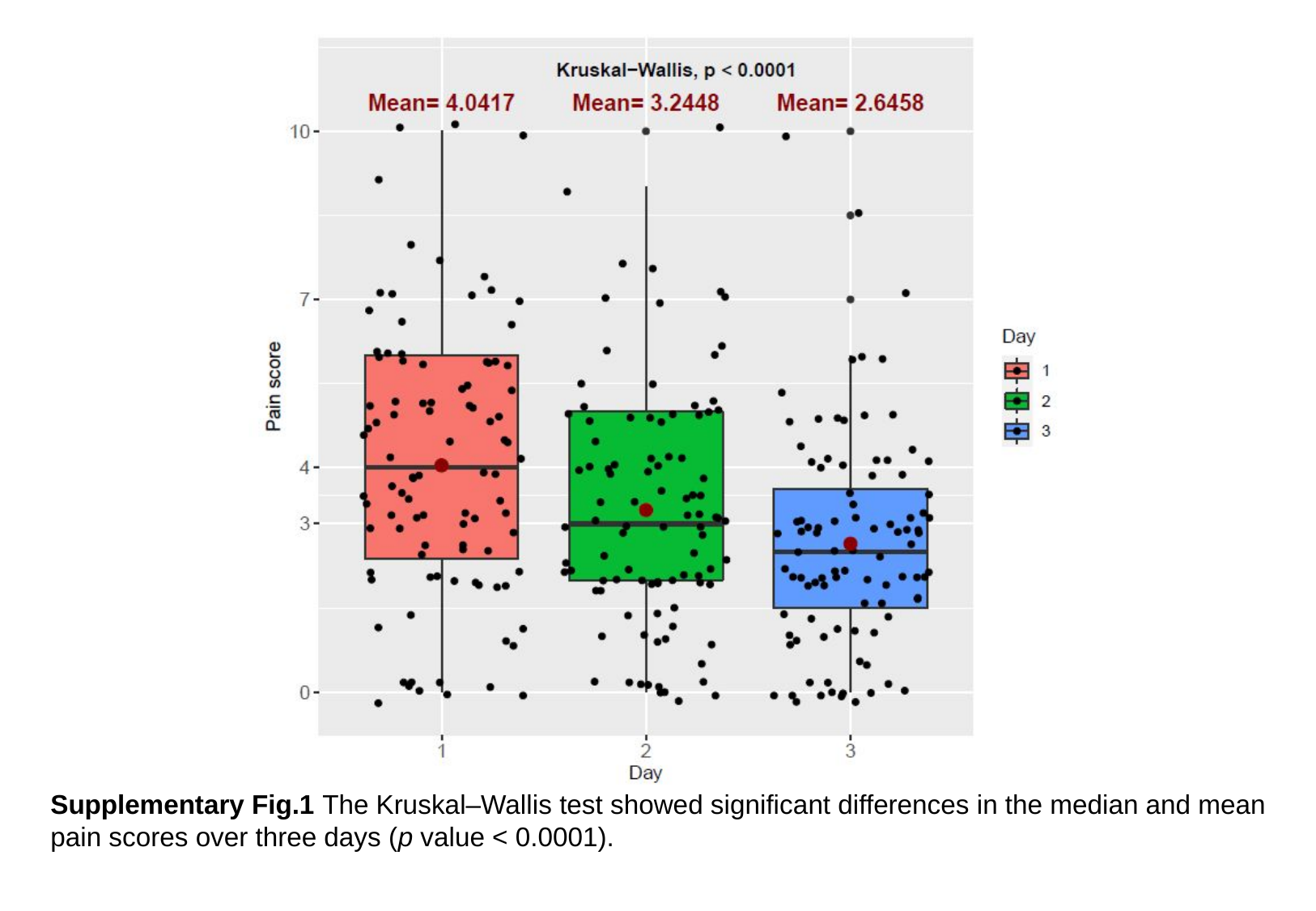

Supplementary Fig.1 The Kruskal‒Wallis test showed significant differences in the median and mean pain scores over three days (p value < 0.0001).
