# Supplementary material for: Lidocaine transdermal patches reduced pain intensity in neuropathic cancer patients already receiving opioid treatment
Source: BMC Palliat Care. 2023 Jan 7;22:4. doi: 10.1186/s12904-023-01126-3 (PMC9824981; doi:10.1186/s12904-023-01126-3)
Supplement: Supplementary file 3 — Additional file 3: Supplementary Fig. 2. Effect on pain relief score and quality of analgesic treatment. A. a five-item pain relief score was assessed on Day 1, Day 2 and Day 3. The results showed no significant difference in the median of the three-day pain relief score (p value = 0.79). B. a five-item quality of analgesic treatment was assessed on Day 1, Day 2 and Day 3. The results showed no significant difference in the median of the three-day (p value = 0.77). [file 12904_2023_1126_MOESM3_ESM.pptx]

## Slide 1
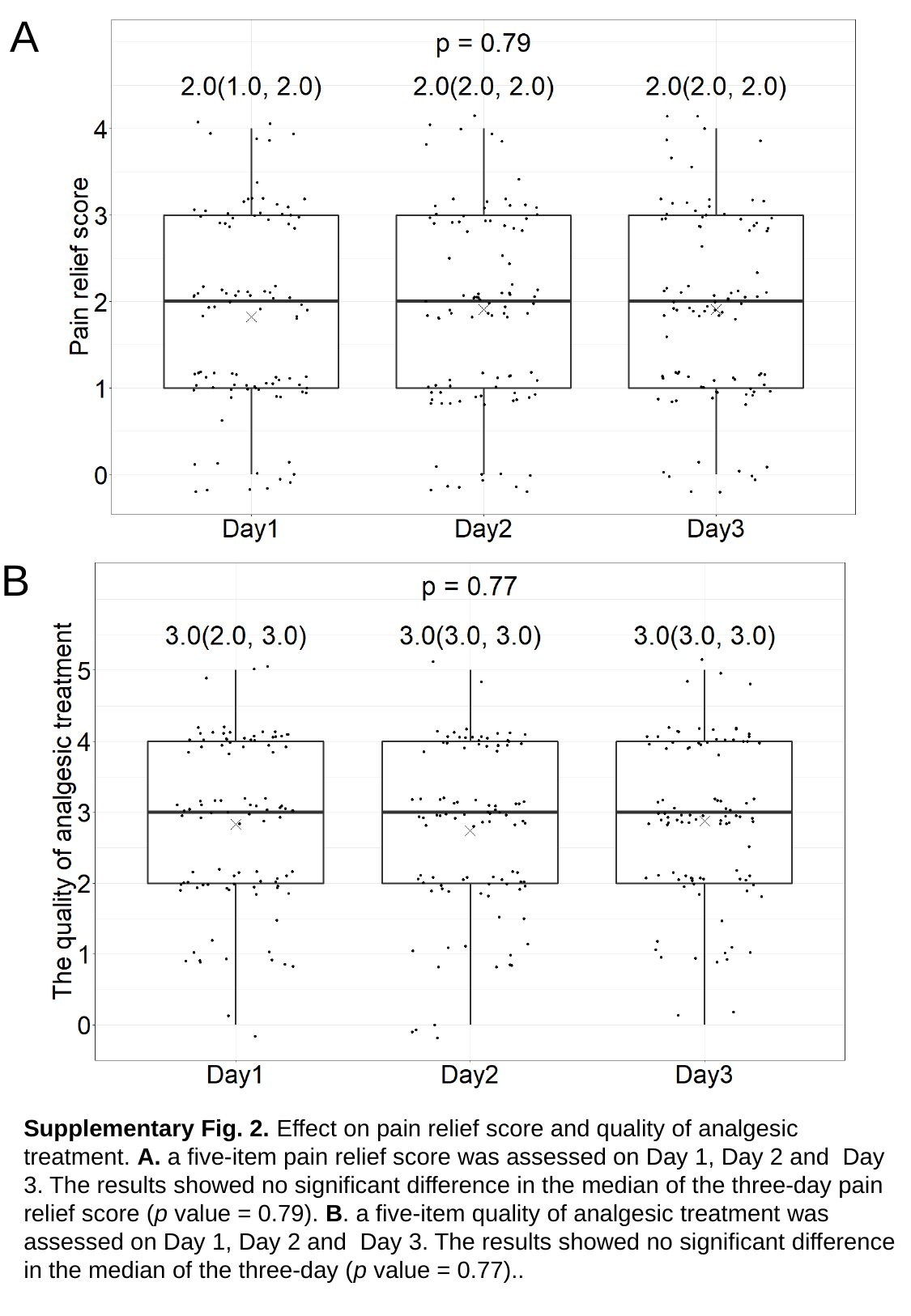

A
B
Supplementary Fig. 2. Effect on pain relief score and quality of analgesic treatment. A. a five-item pain relief score was assessed on Day 1, Day 2 and Day 3. The results showed no significant difference in the median of the three-day pain relief score (p value = 0.79). B. a five-item quality of analgesic treatment was assessed on Day 1, Day 2 and Day 3. The results showed no significant difference in the median of the three-day (p value = 0.77)..
